# Prediction of RC Bridge Member Resistance Decreasing in Time under Various Conditions in Slovakia

**DOI:** 10.3390/ma13051125

**Published:** 2020-03-03

**Authors:** Peter Koteš, Miroslav Strieška, František Bahleda, Petra Bujňáková

**Affiliations:** 1 Department of Structures and Bridges, Faculty of Civil Engineering, University of Žilina, 010 26 Žilina, Slovakia; miroslav.strieska@uniza.sk (M.S.); petra.bujnakova@uniza.sk (P.B.); 2 Laboratory of Civil Engineering, University of Žilina, 010 26 Žilina, Slovakia; frantisek.bahleda@uniza.sk

**Keywords:** member resistance, first-year corrosion rate, corrosion loss, carbon steel, corrosion map, corrosion model

## Abstract

Reliability is one of the most significant requirements for structures given in Eurocodes. Thus, the specific level of safety, serviceability, and durability have to be satisfied to fulfill the reliability of structures. In the case of reinforced concrete (RC) members, the corrosion of reinforcement is not assumed in the stage of structure design, which is in contrast with the structures in service, where the corrosion of reinforcement can significantly decrease their diameter ø in time. In these cases, the moment resistance in time *M_Rd_(t)* decreases during the designed lifetime T_d_ of a structure. The corrosion speed is as a basis for the calculation of moment resistance in time *M_Rd_(t)*, i.e., a first-year corrosion rate *r_corr_* and a corrosion model as well. The corrosion itself is a very complicated issue, so the first-year corrosion rate *r_corr_* and also the corrosion model can be different under various conditions in Slovakia. The paper is focused not only to determine the corrosion speed (first-year corrosion rate *r_corr_* and the corrosion model) and parametric study of the moment resistance in time *M_Rd_(t)* under various conditions in Slovakia but also shows an overview on some parameters that may influence the corrosion process.

## 1. Introduction

All structures shall be designed with an adequate level of reliability. The reliability covers safety, serviceability, robustness, and durability of construction and may be expressed with a reliability margin *G*, see Equation (1), or in the ration form (2). The actual standard approach [1] assumes that the reliability margin *G* has not be changed during the designed lifetime T_d_ of any structural member. It means that the random variable load effects *E* and the random variable member´s resistance *R* have the same level in the time of structure operation, as well as at the end of the designed lifetime of a structure [2]. From that,

(1) G=R−E≥0

or

(2) G=R/E≥1.0

follows, where *G* is the reliability margin, *R* is the random variable resistance, and *E* is the random variable load effects. *R* and E are not usually listed with units (according to [1]), but have a unit depending on the stressing of the element; for instance [kN.m], if the element is subjected to bending, or [kN] if the element is subjected to normal force or shear force, and so on. Consequently, *G* has the same units as *R* and *E* in the case of the differential form (Equation (1)), or *G* is unit-free in the case of the ratio form (Equation (2)).

**Figure 1:**
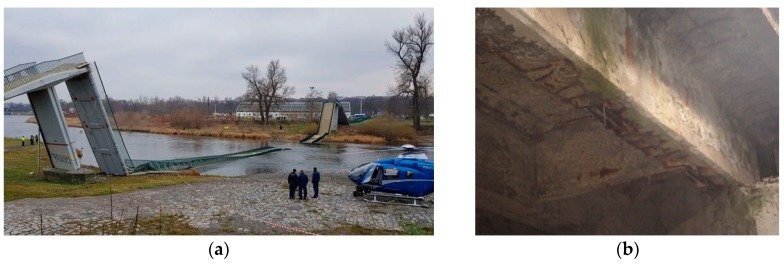
Failures of bridges: (**a**) collapse of the pedestrian bridge due to corrosion of prestressing cables in city Prague, the Czech Republic [3]; (**b**) corrosion of reinforced concrete (RC) T-beam bridge in Kolarovice, Slovakia (2006).

As can be seen in the real structures in service, the random variable resistance *R* is not a constant parameter in time, and many factors may affect it (corrosion, degradation of materials, unexpected cracking, change of static schema, etc.). One of the most general factors from them is the corrosion of the carbon steel, which can lead up to collapse of the structure, for example, as it was in the case of a prestressed pedestrian bridge in the Czech Republic (Figure 1a, [3]). The corrosion of embedded reinforcement in reinforced concrete (RC) members is also a very significant issue; see Figure 1b.

There are many factors that may influence the speed of corrosion, i.e., corrosion depth (the distance between an initial surface of a metal and the nearest concrete surface in time, after corrosion). These factors can be either endogenous such as chemical or physical homogeneity of the surface, composition of the metal, surface treatment or exogenous factors, namely, formation of corrosion products on the surface of metal, time of wetness, air pollution (like CO_2_, *SO*_2_, *Cl*^−^), or climatic data (like temperature, precipitation, wind, etc.). Variability of the factors that affect the corrosion process leads to the various classifications of the corrosion e.g., according to shape of corroding surface (uniform corrosion, pitting corrosion, galvanic corrosion, crevice corrosion), type of the environment (atmospheric corrosion, bacterial corrosion, microbial corrosion, underground corrosion, gaseous corrosion), electrochemical, or chemical corrosion [4,5,6,7]. The most widespread type of corrosion is uniform (or pitting) corrosion under atmospheric conditions’ so-called atmospheric corrosion.

The corrosion of reinforcement does not influence only the resistance (bending, shear) of member—Ultimate Limit States (ULS) [8,9,10], but it also influences the durability of members [11,12] and crack development—Serviceability Limit States (SLS) [13]. The research activities are also focused on the influence of corrosion on mechanical properties of steel reinforcement [14,15].

In the case of RC member, the embedded reinforcement is protected by surrounding concrete during the so-called passive stage (0, *t*_0_). During this stage, the reinforcement does not corrode, and this stage should remain during the entire designed lifetime of a structure. Hence, the corrosion of reinforcement is not taken into account in the design according to EN 1992-1-1 [16] and EN 1992-2 [17], and, if it occurs (so-called active stage (*t*_0_, T_d_)), the reliability margin *G* is automatically decreased in time, so it should be denoted as *G(t)*. Moreover, the random variable load effects are also changed in time *E(t)* due to various impacts (for instance, increase or decrease of action on bridges). Thus, it is possible to define the reliability margin in time *G(t)* by the following equations:

(3) G(t)=R(t)−E(t)≥0

or

(4) G(t)=R(t)/E(t)≥1.0

where *G(t)* is the reliability margin changed in time, *R(t)* is the random variable resistance changed in time, and *E(t)* is the random variable load effects changed in time. Units of *G*, *R*, and *E* are mentioned after Equation (2). 

As was mentioned above, the corrosion may occur on structures during their lifetime, so it is significant to know how the corrosion affects the reliability margin in time *G(t)*. The base for this calculation is the level of the damage of the reinforcement bars in time by corrosion.

The article is focused on the decreasing of the RC member moment resistance in time *M_Rd_(t)* based on the corrosion of the reinforcement. For this reason, the first-year corrosion rate *r_corr_* [μm/year] of carbon steel in various areas of Slovakia, as well as two types of corrosion models, were selected. According to research, it is shown that, in some cases, there is a more correct linear model (in the case of splashing transport mechanism) and power-linear model (in the case of inland areas and spraying transport mechanism) in the paper.

## 2. Change of Moment Resistance in Time Due to Corrosion

The typical structural member of the bridges is a horizontal element (e.g., slab, longitudinal girder, transverse girder, and box girder) primarily subjected to bending and shear (among other stressing due to torsion, fatigue, and so on). The paper is focused on moment resistance *M_Rd_*.

The moment resistance of the RC bridge member according to Eurocode EN 1992-2 [17] outgoing from EN 1992-1-1 [16] is

(5) $$R=M_{Rd}=A_{s1}\cdot f_{yd}\cdot \left[d-\frac{A_{s1}\cdot f_{yd}}{2\cdot b\cdot f_{c}}\right]$$

where

(6) $$A_{s1}=n\cdot \pi \cdot \phi^{2}/4$$

where *M_Rd_* is the ultimate resistance moment of cross-section [kN m], *A*_*s*1_ is the area of longitudinal tensioned reinforcement [m^2^], ø is the initial reinforcement diameter [mm], *n* is the number of reinforcement bars, *f_yd_* is the design value of reinforcement yield strength [N mm^−2^], *d* is the effective depth of the cross-section [m], *b* or *b_eff_* is the effective width of cross-section (slab – *b* = 1.0 m, T-beam – *b_eff_*) [m], and *f_c_* is the design value of the compressive strength of concrete [N mm^−2^]. 

Formulas (5) and (6) do not take into account the degradation of materials in time *t* during the lifetime. The most important degradation parameter changing the material and geometrical parameters of RC cross-section member in time is a reinforcement corrosion, which causes the change of reinforcement diameter *ø(t)* or reinforcement cross-section *A*_*s*1_*(t)* in time *t*. Considering the reinforcement corrosion, formula (6) is changed to form

(7) $$A_{s1}(t)=n\cdot \pi \cdot \phi^{2}(t)/4$$

and formula (5) is transformed into

(8) $$R(t)=M_{Rd}(t)=A_{s1}(t)\cdot f_{yd}\cdot \left[d-\frac{A_{s1}(t)\cdot f_{yd}}{2\cdot b\cdot f_{c}}\right]$$

where *M_Rd_(t)* is the ultimate resistance moment of cross-section changed in time [kN m], *A*_*s*1_*(t)* is the area of longitudinal tensioned reinforcement changed in time [m^2^], *ø(t)* is the initial reinforcement diameter changed in time [mm]. 

From formula (7), it follows that the basic parameter we need to know is information about how the reinforcement diameter *ø(t)* changes in time. Thus, it is needed to determine the model of corrosion losses *D_corr_*. Because the corrosion is a relatively complicated issue, there may be various types of corrosion models. Some of them described below.

The linear model of corrosion loss *D_corr_* recommended for reinforcement in reinforced concrete members, during the active stage, was described by Thoft-Christensen [18]

(9) $$D_{corr}=r_{corr}\cdot t_{as}$$

Or, according to Andrade, Sarria and Alonso [19],

(10) $$D_{corr}=D_{corr}=0.0116\cdot i_{corr}\cdot t_{as}$$

where *D_corr_* is the corrosion loss [µm] (in various literature can be described by various symbols *D*, ∆*ø*, ∆*ø(t)*, *R*, *R_corr_*, *d_corr_*, *x*, *y*, etc.), *r_corr_* is the first-year corrosion rate [μm/year], *i_corr_* is the corrosion current density [μA/cm^2^] (1 μA/cm^2^ is equal to 11.6 μm/year of corrosion, so the convection is $0.0116\cdot i_{corr}=r_{corr}$), and *t_as_* is the length of the active stage, (equal to *t* minus *t*_0_) [years]. 

**Figure 2:**
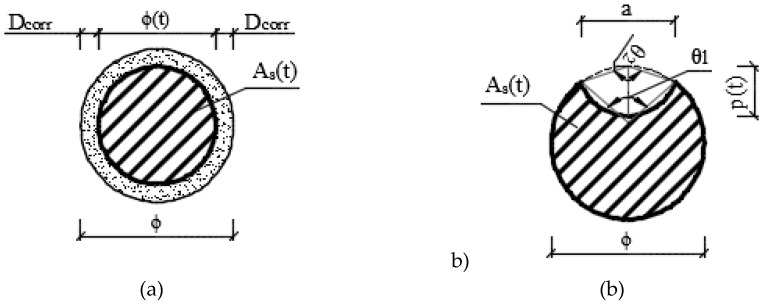
Uniform corrosion of reinforcement bar (**a**) pitting corrosion; (**b**) cross-section.

Thus, the diameter of a reinforcement bar in time *ø(t)*, for uniform corrosion (Figure 2a), can be calculated according to the following equations [18]:

(11) $$\phi \left(t\right)=\phi -2\cdot D_{corr}=\phi -2\cdot r_{corr}\cdot t_{as}=\phi -2\cdot r_{corr}\cdot \left(t-t_{0}\right)$$

or, according to [19],

(12) $$\phi \left(t\right)=\phi -2\cdot D_{corr}=\phi -2\cdot 0.0116\cdot \left(t-t_{0}\right)\cdot i_{corr}=\phi -0.0232\cdot \left(t-t_{0}\right)\cdot i_{corr}$$

where *ø* is the initial reinforcement diameter [mm], *ø(t)* is the reinforcement diameter changed in time [mm], *t* is the exposure time of structure [years], and *t*_0_ is the length of the passive stage [years]. 

Power function model according to [20] assumes the corrosion on the flange of the I-shape of a structural steel member

(13) $$D_{corr}=r_{corr}\cdot t_{as}^{A_{1}}$$

where *A*_1_ is the constant [–]. 

The above-mentioned power model of corrosion is recommended for structural steel members. Since structural steel and reinforcement are both carbon steels (according to EN 9224 [21]), it is assumed that the power model can also be used for reinforcement in some cases as shown later.

Moreover, the last significant model according to the actual standard approach [21] has the combination of the previous two models. The functions of the power-linear model are

(14) $$D_{corr}(t_{as}\leq 20)=r_{corr}\cdot t_{as}^{b}$$

(15) $$D_{corr}(t_{as}>20)=r_{corr}\cdot \left[20^{b}+b\cdot (20^{b-1})\cdot (t_{as}-20)\right]$$

where *b* is the metal-environment-specific time exponent for steel [–]. 

This corrosion model assumes the creating of the corrosion products on the surface of the material that protects the original metal and slows down the corrosion losses. This protective layer, during the first twenty years represented by a power function of the corrosion loss *D_corr_*(*t_as_* ≤ 20) (14), then follows the linear function of the corrosion loss *D_corr_*(*t_as_* > 20) (15). The exponent *b* can be calculated either by a chemical composition of a metal or the recommended value for carbon steel is *b* = 0.523. In both cases, the value of metal-environment-specific time exponent *b* is closer to 0.5 rather than to 1.0, and this exponent may be increased by ∆*b* in areas with a higher concentration of chloride deposition rate *Cl*^−^ [21] described by equation

(16) $$\Delta b=0.0845\cdot {\left[Cl^{-}\right]}^{0.26}$$

where ∆*b* is the increasing of the exponent *b* [–], and *Cl*^−^ is the chloride deposition rate [mg/(m^2^·day)]. 

Now, it is possible to modify Equation (8), taking into account the linear corrosion model, into final form for moment resistance [22] depending only on time

(17) $$M_{Rd}(t)=M_{Rd}(0)+k_{m1}\cdot \left(t-t_{o}\right)+k_{m2}\cdot {\left(t-t_{o}\right)}^{2}+k_{m3}\cdot {\left(t-t_{o}\right)}^{3}+k_{m4}\cdot {\left(t-t_{o}\right)}^{4}$$

For more information and a description of each variable, see [22]. The model takes into account the change of reinforcement diameter *ø(t)* (decreasing) in time using *r_corr_* or *i_corr_*, which are reflected in the parameters *k*_*m*1_ to *k*_*m*4_.

Moment resistance of a cross-section in time *M_Rd_(t)* described by the power-linear function of the corrosion loss *D_corr_*, and by the modifying of formula (8) is given by the following formulas:

(18) $$M_{Rd}\left(t\leq 20\right)=M_{Rd}\left(0\right)+k_{m1}.{\left(t_{as}\right)}^{b}+k_{m2}.{\left(t_{as}\right)}^{2.b}-k_{m3}.{\left(t_{as}\right)}^{3.b}-k_{m4}.{\left(t_{as}\right)}^{4.b}$$

(19) $$M_{Rd}\left(t\;>\;20\right)=k_{m5}+k_{m6}.(t^{*})+k_{m7}.{\left(t^{*}\right)}^{2}+k_{m8}.{\left(t^{*}\right)}^{3}+k_{m9}.{\left(t^{*}\right)}^{4}$$

where

(20) $$t^{*}=t-t_{0}-20\;\left[years\right]$$

(21) $$M_{Rd}(0)=A.\phi^{2}-B.\phi^{4}$$

where *k*_*m*1_–*k*_*m*9_ are the parameters depending on materials’ and geometrical’ characteristic in time, *A*, *B* are the parameters constant in time

(22) $$k_{m1}=-4\cdot A\cdot \phi \cdot r_{corr}+8\cdot B\cdot \phi^{3}\cdot r_{corr}$$

(23) $$k_{m2}=4\cdot A\cdot r_{corr}^{2}-24\cdot B\cdot \phi^{2}\cdot r_{corr}^{2}$$

(24) $$k_{m3}=32\cdot B\cdot \phi \cdot r_{corr}^{3}$$

(25) $$k_{m4}=16\cdot B\cdot r_{corr}^{4}$$

(26) $$k_{m5}=A\cdot {\left(\phi -2\cdot r_{corr}\cdot 20^{b}\right)}^{2}-B\cdot {\left(\phi -2\cdot r_{corr}\cdot 20^{b}\right)}^{4}$$

(27) $$k_{m6}=4\cdot r_{corr}\cdot b\cdot 20^{b-1}\cdot \left[2\cdot B\cdot {\left(\phi -2\cdot r_{corr}\cdot 20^{b}\right)}^{3}-A\cdot \left(\phi -2\cdot r_{corr}\cdot 20^{b}\right)\right]$$

(28) $$k_{m7}=A\cdot {\left(2\cdot r_{corr}\cdot b\cdot 20^{b-1}\right)}^{2}-6\cdot B\cdot {\left(\phi -2\cdot r_{corr}\cdot 20^{b}\right)}^{2}\cdot {\left(2\cdot r_{corr}\cdot b\cdot 20^{b-1}\right)}^{2}$$

(29) $$k_{m8}=4\cdot B\cdot {\left(2\cdot r_{corr}\cdot b\cdot 20^{b-1}\right)}^{3}\cdot \left(\phi -2\cdot r_{corr}\cdot 20^{b}\right)$$

(30) $$k_{m9}=B\cdot {\left(2\cdot r_{corr}\cdot b\cdot 20^{b-1}\right)}^{4}$$

(31) $$A=\frac{\pi \cdot f_{yd}\cdot n\cdot d}{4}$$

(32) $$B=\frac{\pi^{2}\cdot f_{yd}^{2}\cdot n^{2}}{32\cdot b\cdot f_{cd}}$$

The models take into account only the increase of the rust volume and the reduction of the cross-section of the reinforcement, but do not take into account the spalling of the concrete cover. To do any calculation of moment resistance changing in time *M_Rd_(t)*, it is necessary to know the values of corrosion rate *r_corr_* as an input parameter.

## 3. First-Year Corrosion Rate *r_corr_* of Carbon Steel

The standard EN ISO 9223 [23] describes the designation of the yearly corrosion rate for the first year of exposure by two methods. The first method is based on a determination of the first-year corrosion rate *r_corr_* using measurements on standard specimens (specimens under outdoor environment). The second method is based on the determination of the first-year corrosion rate *r_corr_* using environmental information obtained from a net of meteorological measurement stations and using the dose–response functions (see paragraph 3.1). The first method is very time and money consuming, so the second method is preferred and used in a general way.

### 3.1. Dose–Response Functions According to the Actual Standard Approach

There have been many dose–response functions of carbon steel created during the years; see [24]. Nowadays, the actual standard [23] describes the dose–response functions for standard materials like carbon steel, zinc, copper, and aluminum. These functions have been based on climatic parameters like temperature T, relative humidity Rh, Sulphur dioxide *SO*_2_, and chloride deposition rate *Cl*^−^. The following Equation (33) describes the first-year corrosion rate *r_corr_* of carbon steel

(33) $$\begin{array}{ll} r_{corr} & =\;1.77\cdot {\left[SO_{2}\right]}^{0.52}\cdot \exp(0.02\cdot Rh+f_{CS})+0.102\cdot {\left[Cl^{-}\right]}^{0.62}\cdot \\ & \cdot \exp(0.033\cdot Rh+0.04\cdot T) \end{array}$$

where *SO*_2_ is the deposition rate of Sulphur dioxide [mg/(m^2^·day)], *T* is the temperature [°C], Rh is the relative humidity [%], *Cl*^−^ is the chloride deposition rate [mg/(m^2^·day)], and *f_CS_* = 0.15·(*T*–10) when *T* < 10 °C; otherwise, *f_CS_* = –0.054·(*T*–10). 

This first-year corrosion rate *r_corr_* is the base parameter for calculation of corrosion loss *D_corr_* in time (in unit [μm]) according to standard [21] described in Section 2.

The Slovak Hydrometeorological Institute (SHMI) performs measurements of all the input parameters. The parameters like temperature and relative humidity are measured as is recommended in the standard EN 9225 [25] (approximated in 120 stations in Slovakia), while sulphur dioxide is measured as a concentration in the air in unit [µg/m³] (in about 50 stations). Thus, the convection between deposition rate of Sulphur dioxide *SO*_2_ and concentration of Sulphur dioxide in the air *SO*_2,air_ is equal to [23]

(34) $$\begin{array}{ll} SO_{2}\left[mg/m^{2}\cdot d\right] & =0.8\cdot SO_{2,\mathrm{air}}\left[\mu g/m^{2}\right]\\ & \to SO_{2,\mathrm{air}}\left[\mu g/m^{2}\right]=\frac{SO_{2}\left[mg/m^{2}\cdot d\right]}{0.8} \end{array}$$

The chloride ions in the air *Cl*^−^_air_ in unit [mg/L] are measured in only five stations in Slovakia (in rural or industrial environment, not near roads), which is a very small number for the creation of corrosion maps (see Section 3.3), as well as there being no standard recommended convection between chloride ions in the air *Cl*^−^_air_ (in unit [mg/L]) and chloride deposition rate *Cl*^−^ (in unit [mg/(m^2^·day)]). Chloride deposition rate *Cl*^−^ has not been measured in Slovakia, so they were taken from literature and code (Section 3.5 focuses on this matter).

### 3.2. Long-Term Monitoring of the First-Year Corrosion Rate

**Figure 3:**
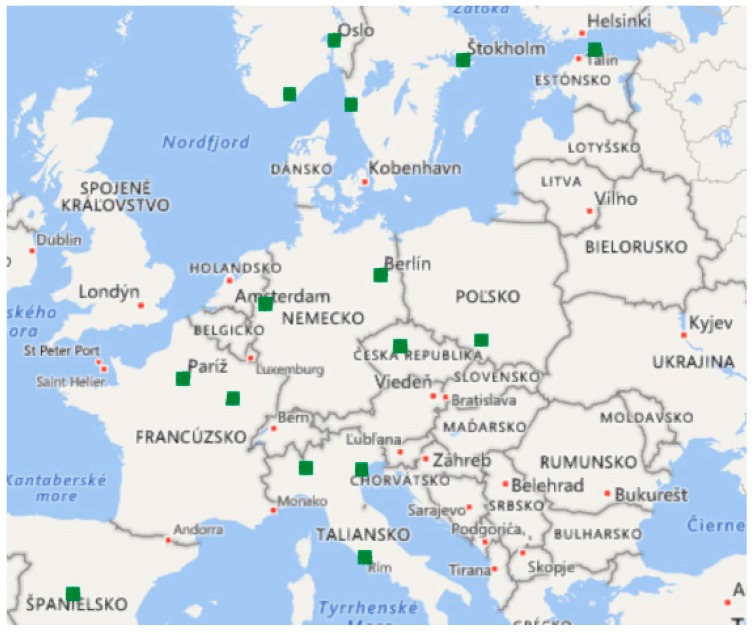
Locations of measurement stations and exposure stations in the project of IPC Materials [26].

The first-year corrosion rate *r_corr_* is changing in various areas, as well as during the years. Thus, it is significant to know since this corrosion rate should be observed for the correct determination of how high the corrosion rate *r_corr_* in various areas in Slovakia is. The program IPC Materials–International Co-operative Program on the Effect on Materials including Historic and Cultural Monuments has been monitoring the air pollution and corrosion effect in various areas of Europe (the Czech Republic, Germany, Italy, Norway, Sweden, Spain, Finland, France, and Poland) since 1985 (see Figure 3) [26].

**Figure 4:**
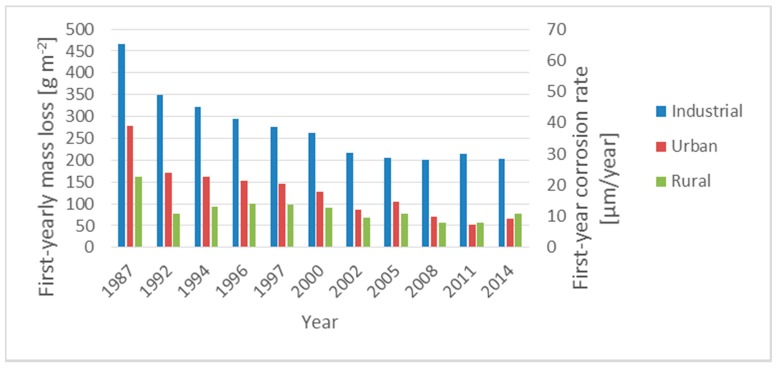
Average first-year mass loss ML and first-year corrosion rate of carbon steel *r_corr_* from 1987.

Figure 4 presents the average first-year corrosion effect (first-year corrosion rate *r_corr_* and first-year mass loss ML) observed in this research. It can be seen that the corrosion rate *r_corr_* has not been changed roughly since 2002. Thus, the creating of the corrosion maps (see Section 3.3), for determining the first-year corrosion rate *r_corr_* in Slovakia, can be sufficient since 2004.

### 3.3. Corrosion Maps of Carbon Steel

The corrosion maps represent, in graphical form, the first-year corrosion rate *r_corr_* calculated according to Equation (33) in various areas of the Slovak Republic. The input parameters like temperature, relative humidity, and Sulphur dioxide were obtained from co-operation with the Slovak Hydrometeorological Institute (SHMI). The chloride deposition rate, in inland areas, is a relatively small value, usually under 3 mg/(m^2^·day), so their effect is neglected in creating corrosion maps. Only chlorides calculated from input data from SHMI were taken into account in this part. The chloride from de-icing agents are considered in Section 4.

The process of creating corrosion maps has several steps, and programs like Microsoft Excel, Surfer, QGIS, and GIMP were used. The program Surfer extrapolates all the input parameters, to the point without knowing parameters, to about 93,000 points of results on a grid of 1 × 1 km to all of Slovakia by the formula

(35) $$Z_{A}=\sum_{i=1}^{n}W_{i}\cdot Z_{i}$$

where *Z_A_* is the estimated value as a linear combination of *Z_i_* and *W_i_*, *n* is the number of neighboring measured values, *Z_i_* is the measured value at the *i*-th location, and *W_i_* is the weight factor that calculates by finding the semi-variogram values between distances of all input points and output pixels. 

**Figure 5:**
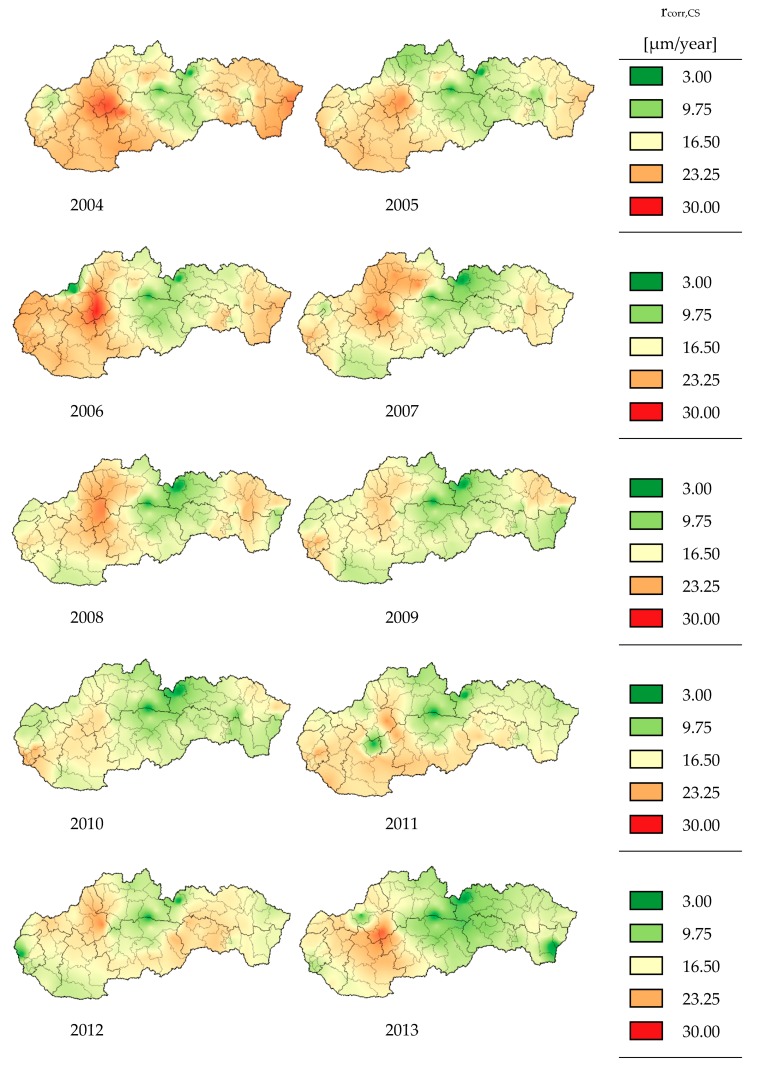

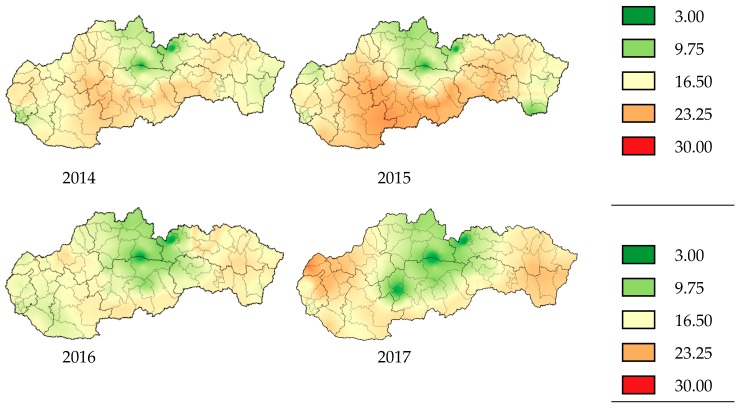
First-year corrosion rate of carbon steel *r_corr_* from 2004 to 2017.

The corrosion maps created according to this process from 2004 to 2017 are shown in Figure 5. The scale of bright tones was chosen from the minimum value of the calculated first-year corrosion rate (*r_corr_* = 3.00 µm/year) to the maximum value of the calculated first-year corrosion rate (*r_corr_* = 30.00 µm/year).

### 3.4. Impacts of Input Parameters on Corrosion Maps

It may raise the question of which factor affects the most corrosion rate *r_corr_* in various areas in Slovakia. As can be seen on the maps of environment aggressiveness of Slovakia [27], the maps of the concentration of Sulphur dioxide in air *SO*_2,air_ have the most similar areas of concentration as maps of the first-year corrosion rate. 

**Figure 6:**
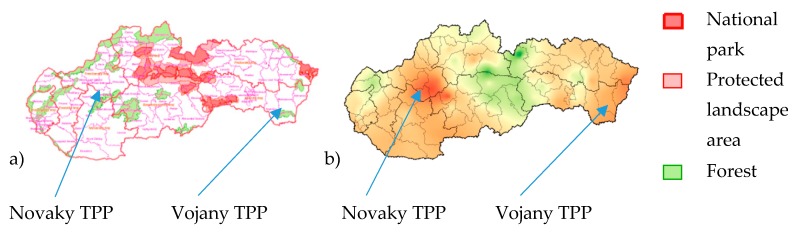
Protected territories in Slovakia (**a**), the corrosion rate of carbon steel in 2004 (**b**).

The sources of Sulphur dioxide *SO*_2,air_ can be either a human source or natural source. The primary human source of Sulphur dioxide is by burning of fossil fuels such as coal and oil. Two thermal power plants are located in the Slovak Republic, in Nováky and Vojany, information on [28]. In addition, the areas of National park have a lower corrosion rate; see Figure 6.

**Figure 7:**
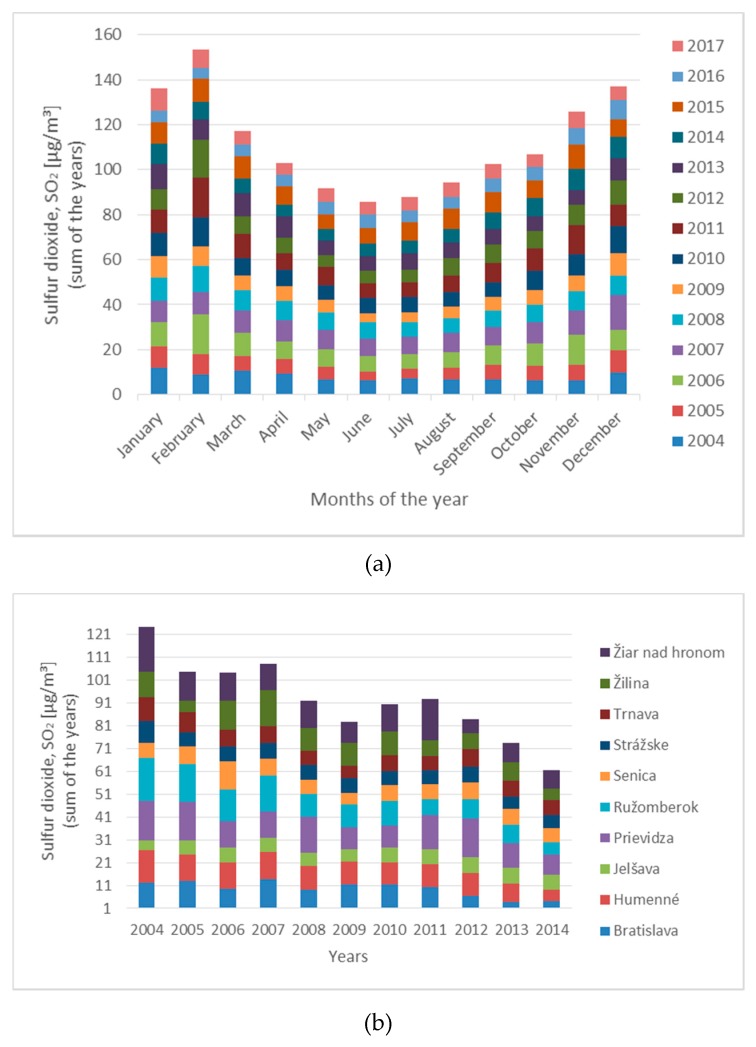
Sum of the monthly values of *SO*_2,air_ concentration from 2004 to 2017; (**a**) and sum of values of sulfur dioxide concentration *SO*_2,air_ in some selected locations in Slovakia (**b**).

For the variation of the sulphur dioxide concentration in the air *SO*_2,air_ during the year (in particular months of the year), see Figure 7. The vertical axis (in Figure 7) represents the sum of year average concentration of sulphur dioxide in Slovakia from 2004 to 2017, on which it can be seen that this concentration increased during the winter seasons (November to March), and it should be the result of the higher burning of fossil fuels in thermal power plants and in houses (heating houses in villages with coal and wood). On the side, the minimal concentration is during the summer season. The average value of *SO*_2_ decrease in time in Slovakia, as it can be seen from measured values given in Figure 7b).

The most significant natural resource is a volcanic eruption. The most massive volcanic eruption was in April 2010 and in May 2011, which paralyzed almost all air traffic transport over Europe. Despite this fact, the concentration of *SO*_2,air_ was not significantly increased in these years. The values of *r_corr_* were calculated according to formula (33), which also takes into account chloride ions.

### 3.5. Effect of Chloride Deposition Rate

The higher concentration of chloride deposition rate *Cl*^−^ increases the first-year corrosion rate *r_corr_*, as well as changes the corrosion model, for the first twenty years (see formula (14)) to the more linear function by the increasing of exponent *b* by ∆*b* (see Equation (16)). It may have a very significant effect on the speed of corrosion and on the decreasing of the reliability in time as well.

**Figure 8:**
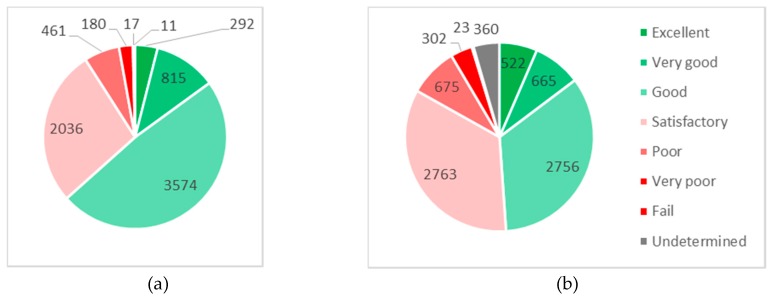
Grading system of the bridge under SRA in 2004 (**a**) and in 2017 (**b**).

Here, it can distinguish three possible areas of salinity. The first is valid in areas under 3 mg/(m^2^·day) for which the corrosion maps in Section 3.3 and corrosion model without increasing the exponent *b* by ∆*b* are valid. The other two, where the chloride deposition rate is significantly higher, i.e., in areas where de-icing salt on the road infrastructure network in winter seasons is applied either by spraying transport of de-icing salt (in some winter seasons *Cl*^−^ = 90 mg/(m^2^·day)) or by splashing transport of de-icing salt (in some winter seasons *Cl*^−^ = 8000 mg/(m^2^·day)) [29,30,31]. This effect can be seen mainly on the structures around the road and bridges, e.g., abutments, piles, and superstructure of bridges. Figure 8 shows the grading system of the bridge, under Slovak Road Administration (SRA), in 2004 and in 2017, where it can be seen that the reliability of the bridges is decreased during their designed lifetime, information on Slovak Road Administration [32].

For determination of the corrosion model, the value of the exponent *b* + ∆*b* should be known. Some research describes this value over one, up to the value *b* = 1.79 [33,34,35] and another one under value one [36].

For the parametric study, in the case of spraying transport mechanisms of de-icing salt, the exponent *b* was chosen *b* + ∆*b* ≈ 1 by three facts: the first, the standard EN 9224 [21], recommends that the value of the metal-environment-specific time exponent *b* + ∆*b* is usually under one; the second, the results of the accelerated Neutral Salt Spray (NSS) test, according to EN 9227 [37], confirms the value of exponent *b* close to one (*b* = 1.08, see Equation (31) and Table 1) and not *b* + ∆*b* = 1.211 as it shall be calculated according to Equation (13), when the deposition rate of chloride in the corrosion chamber is *Cl*^−^ = 4.290 mg/(m^2^·day). The NSS tests were made in a laboratory of the University of Zilina on specimens of reinforcement of diameters ø6, ø10, ø14, and ø25 (10 specimens of each diameter means 40 specimens) using corrosive chamber type DCTC 1200 P. The corrosive losses on laboratory specimens were calculated using formula (36), which was modified from formula (14)

(36) $$D_{ch}=r_{ch,first}\cdot {\left(t_{ch}\right)}^{b_{ch}}$$

where *D_ch_* is the corrosion loss of non-protected carbon steel samples (reinforcement bar of diameters ø6, ø10, ø14, and ø25, and length 300 mm) in the corrosion chamber [µm], *r_ch,first_* is the first-daily corrosion rate in the corrosion chamber [µm/day], *t_ch_* is the time of closed corrosion chamber [days], and *b_ch_* is the exponent related to the corrosion model in the corrosion chamber [–]. 

The third is the RC T-beam bridge under service in Kolárovice village has a corrosion model very close to the linear model (i.e., *b* + ∆*b* ≈ 1) (see [38]).

## 4. Parametric Study of Moment Resistance in Time under Various Conditions in Slovakia

For the parametric study, two types of corrosion models were chosen. The first is a power-linear model that represents the areas where the de-icing salt is not applied or is applied by spraying transport mechanisms, while the second model represents the areas where the de-icing salt is applied by splashing transport mechanisms and thus the corrosion model approximated to the linear function. By this, the moment resistance of RC bridge member *M_Rd_(t)* in time was derived as a power-linear decrease in the moment resistance in time using Equations (18) and (19) and a linear decrease in the moment resistance in time using formula (17). No pit corrosion model was used as the surface corrosion was verified experimentally on the bridge [38].

**Figure 9:**
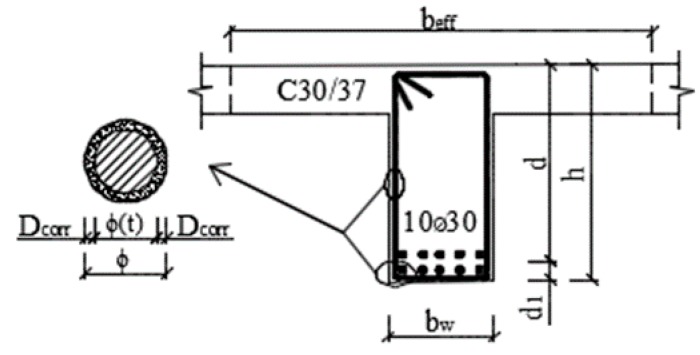
Cross-section of the RC T-beam bridge in Kolárovice village.

For the parametric study of the moment resistance *M_Rd_(t)* in time was chosen, the dimensions of the real RC T-beam bridge in Kolárovice village; see Figure 9. The bridge is the reinforced concrete single span beam bridge. The superstructure consists of bridge slab and of six main beams. The transverse load distribution is ensured by transverse beams. More information about the bridge is possible to see in [38]. All dimensions and material´s characteristics were measured on real structures using destructive or non-destructive testing. The concrete strength was also tested using a Schmidt hammer. The design documentation was not available, and the original values of the material strengths were not known, so it was not possible to compare them with the measured values. The design documentation was not available, and the original values of the material strengths were also not known, so it was not possible to compare them with the measured values. Thus, only the measured geometric and material properties of the bridge were taken into account in the parametric study.

Table 2 shows the input parameters used in the parametric study of the RC T-beam cross-section. As already mentioned, three conditions of salinity were chosen.

The first represents areas where chloride deposition rate *Cl*^−^ is as in inland areas, under 3 mg/(m^2^·day). In this case, the chloride ions affect the corrosion rate *r_corr_* not so significantly, the minimum *r_corr_* was increased from 3 μm/year to 5.4 μm/year and the maximum *r_corr_* from 30 μm/year to 33.64 μm/year (see Figure 5), and the ∆*b* was increased by 0.112 in comparison when the chloride deposition rate is zero or 3.00 mg/(m^2^·day). As was mentioned above, the deposition rate of chloride has not been measured in Slovakia so, for this case, it was neglected in the creation of corrosion maps and parametric study, as well.

The more significant influence on first-year corrosion rate *r_corr_* has spraying transport mechanisms of de-icing salt on structures. This transport mechanism represents the second conditions under which the structure may occur. For this reason, the average yearly concentration of chloride chose *Cl*^−^ = 11.20 mg/(m^2^·day) [29]. Then, the corrosion rate *r_corr_* was increased from 3.00 μm/year to 10.12 μm/year and from 30 μm/year to 38.00 μm/year; see Table 2.

Finally, the parameters for splashing transport mechanisms of de-icing salt were calculated, see Table 2. In this case, the value of *r_corr_* (according to formula (33)) and exponent *b* + ∆*b* (according to formula (16)) are several times higher than in the previous two cases.

Long-term experimental research is being carried out on the bridge structure, measuring the actual corrosion losses (*r_corr_*). The first measurement of corrosion losses was made in 2005, the next in 2015, 2016, 2017, and 2018 (the measurement in 2019 are not included). From the measurements, the yearly corrosion rates follow:

- *r_corr_* = 130.38 µm/year (2005–2015),
- *r_corr_* = 169.40 µm/year (2015–2016),
- *r_corr_* = 204.26 µm/year (2016–2017),
- *r_corr_* = 183.78 µm/year (2017–2018).

A splashing transport mechanism on the bridge due to poor drainage solutions (the same level of pavement and cornices causes melted snow to flow with chloride ions from de-icing salts during the winter season) was seen. The measurement of real corrosion losses confirms the legitimacy of using the minimum and maximum values considered in a parametric study (*r_corr, min_* = 81.41 μm/year, *r_corr, min_* = 158.88 μm/year) because the real corrosion losses may be greater.

**Figure 10:**
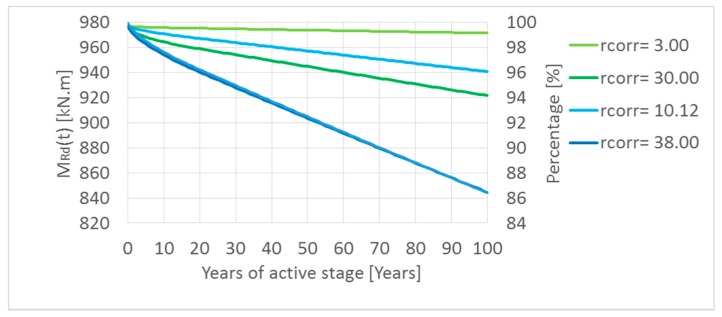
Decreasing of the moment resistance in time *M_Rd_(t)* by corrosion of reinforcement in the case of inland areas (green-lines) and spraying transport mechanisms of de-icing salt (blue-lines) in Slovakia.

**Figure 11:**
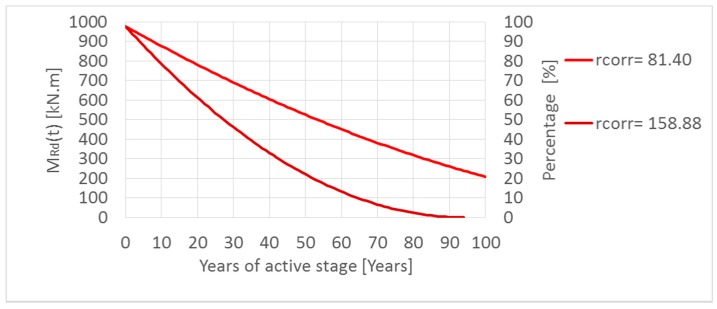
Decreasing of the moment resistance in time *M_Rd_(t)* by splashing transport mechanisms of de-icing salt in Slovakia.

A parametric study was done taking into account minimum and maximum calculated first-year corrosion rate *r_corr_* in Slovakia since 2004. The results are shown in Figure 10 and Figure 11. In the parametric study, for simplifying, the length of the passive stage (*t*_0_) was neglected, so it means that the reinforcement corrosion starts at the beginning. By means of parametric study, the main aim was to express the influence of reinforcement corrosion on a possible decrease of resistance of the element—by neglecting the passive stage, extreme values were obtained. The length of the passive stage depends mainly on the aggressiveness of the environment and the thickness of the covering layer, which in practice can reach values of about 10 to 40 years. However, in extreme winter conditions and very poor maintenance, the passive stage can be reduced to only about 5–15 years [2,22,27]. For this reason, firstly, the passive stage in the parametric study was neglected.

Figure 10 shows that decreasing of the moment resistance in time *M_Rd_(t)* for the RC T-beam bridge, with the cross-section described above, is relatively very low (from 99.40% to 94.30% of the basic initial value of *M_Rd_(t)* in time *t* = 0), which represents inland areas where the salinity is very low (green lines in the graph).

In the case of spraying transport mechanisms of de-icing salt, the moment resistance after one hundred years of corrosion of reinforcement has been decreased by only 4% in areas where the first-year corrosion rate *r_corr_* is lower by 14% (up to 86%) in areas with the upper value of *r_corr_*; see Figure 10 (blue lines) in the graph.

In the case of splashing transport mechanism, the moment resistance has been significantly decreased (Figure 11). Only in the first fifty years of the corrosion (active stage *t_as_*) was the moment resistance 60% or 24% depending on considered values *r_corr_*. If the reinforced concrete member has been projected at 80% of their capacity (*G* = 0.8), conditions (3) and (4) of the reliability margin in time *G(t)* will be not satisfied in the first eleven or twenty years of the active stage depending on Slovak’s locality.

**Figure 12:**
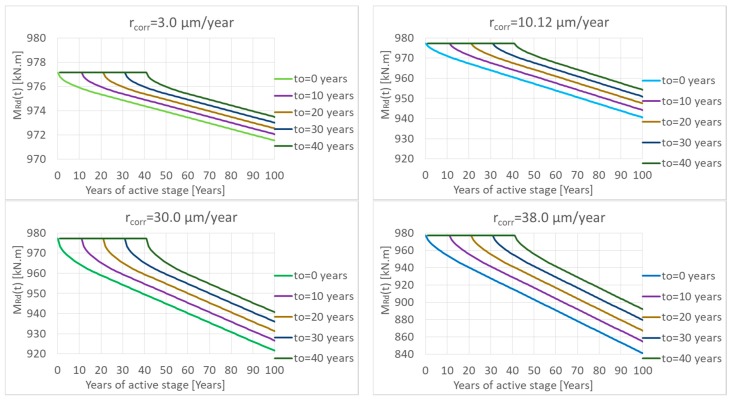

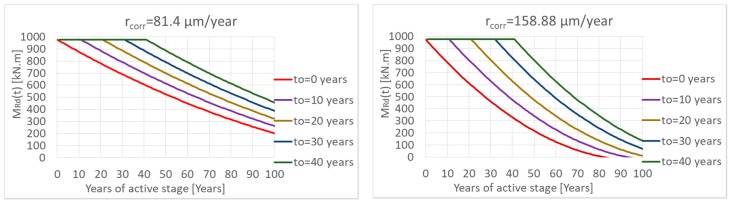
Decreasing of the moment resistance in time *M_Rd_(t)* by corrosion of reinforcement taking into account the passive length (10, 20, 30, and 40 years).

In the next parametric study, the protection of the reinforcement in concrete with a concrete cover was considered, i.e., the passive stage was taken into account. Many models for calculation of the passive stage length exist. This paper is not focused on the precise calculation of the passive stage, so the models are not presented. The length of passive stage for parametric study was only assumed in lengths 10, 20, 30, and 40 years (as was mentioned above). The results are presented in Figure 12. 

**Figure 13:**
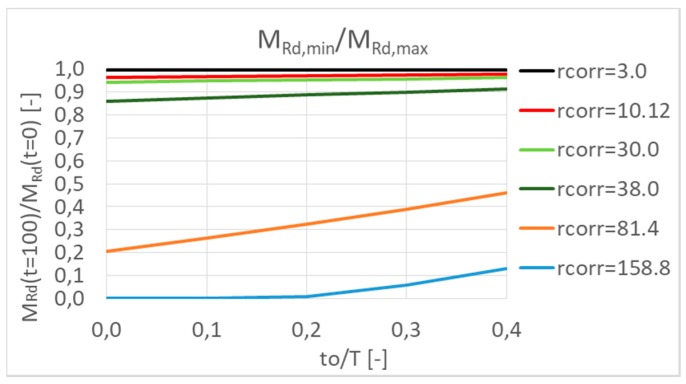
Influence of the passive stage length on the minimum moment resistance in time *M_Rd_(t)*.

From the results, it follows that the length of passive stage has a higher influence in the case of higher values of *r_corr_*. Because of lower *r_corr_* values, there is also a smaller decrease in overall resistance. Increasing the passive stage length reduces the decrease in resistance by about 1% to 5%. In the case of larger *r_corr_* values, a longer passive stage length reduces the loss of resistance by up to 13–26% (see Figure 13).

According to various literature work, pitting corrosion has a greater effect on the change in resistance than surface corrosion due to the higher rate expressed by the *R_corr_* parameter, which takes values from 2 to 8 [39].

In the case of pitting corrosion, the most frequently considered shape of a pit is semi-spherical (see Figure 2b), and the pitting penetration *p(t)* is equal to

(37) $$p(t)=\left(t-t_{0}\right)\cdot R_{corr}\cdot r_{corr}$$

or

(38) $$p(t)=0.0116\cdot \left(t-t_{0}\right)\cdot R_{corr}\cdot i_{corr}$$

where *R_corr_* is the coefficient takes into account pitting corrosion [–] and its value can be from 2 to 8 [39], θ_1_ is the angle in the case of pitting corrosion [°], and θ_2_ is the angle in the case of pitting corrosion [°]. 

**Figure 14:**
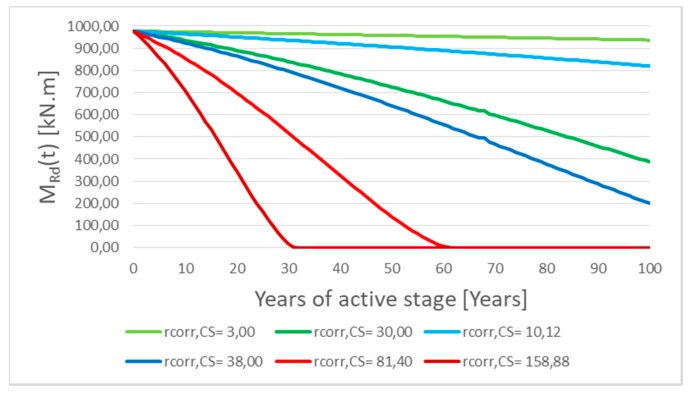
Decreasing of the moment resistance in time *M_Rd_(t)* taking into account the pitting corrosion.

The results take into account the pitting corrosion with *R_corr_* = 4 chosen as shown in Figure 14.

The results show the expected greater impact of pitting corrosion than surface corrosion. In the case of bridge structures, which are typically in a more aggressive environment with a higher *r_corr_* value as building structures, the service life of the structure may only be about 30–40 years unless the passive stage is considered; with a passive stage, the life span may be about 40–90 years.

## 5. Conclusions

The article is focused on the decreasing of the moment resistance in time *M_Rd_(t)* of the reinforced concrete T-beam bridge. The first-year corrosion rate of carbon steel *r_corr_* was calculated in various areas in Slovakia using dose–response functions. Moreover, represented values of *r_corr_* were used for the creation of corrosion maps of carbon steel in Slovakia during the time period from 2004 to 2017. On the basis of this first-year corrosion rate *r_corr_* and the research in areas with a higher concentration of chloride deposition rate *Cl*^−^, the parametric study was done. Three conditions were simulated under which the T-beam cross-section of the bridge may occur, i.e., inland areas (*Cl*^−^ < 3.00 mg/(m^2^·day)), spraying transport mechanisms of de-icing salt (*Cl*^−^ = 11.20 mg/(m^2^·day), or splashing transport mechanisms of de-icing salt where yearly deposition rate of chloride is *Cl*^−^ ≥ 760.50 mg/(m^2^·day) (see Table 2 – splashing transport mechanism). The conditions take into account a more aggressive environment along the road network, which is not able to take into account using the values measured by SHMI. The paper focuses mainly on the effect of reinforcement corrosion on the possible reduction of the moment resistance of the element in time—the passive stage was neglected. For this reason, the model neglects the influence of permeability of concrete, concrete cover thickness, pH factor of concrete, and carbonation—these parameters were not implemented in the model. Thus, possible extreme resistance values of RC elements were obtained. The model may take into account different levels of corrosion, e.g., also increased values due to a crack in the cover layer.

The results show:

- the decreasing of the moment resistance in time *M_Rd_(t)* is very significant in the case of splashing transport mechanisms of de-icing salt,
- only after twenty years, in areas with lower first-year corrosion rate, or, after eleven years, in areas with a higher first-year corrosion rate in Slovakia; of the active stage *t_as_*, the T-beam cross-section is 80% of their capacity,
- pitting corrosion has a higher influence on decreasing of the moment resistance in time *M_Rd_(t)* in time—the service life of the bridge structure can be between one-third and two-thirds of the design life (not considering passive stage). If the passive stage is considered, the service life can be more than half of the design lifetime.
- the corrosion maps and the parametric study also confirm that the different aggressive conditions in Slovakia affect the moment resistance in time *M_Rd_(t)*.
- areas with less first-year corrosion rate *r_corr_* are around the National park and areas with higher corrosion rate are e.g., around the town Nováky.
- thus, if it is the same structure built in various localities in Slovakia, the moment resistance in time *M_Rd_(t)* may be decreased from 99.40% to 94.30% in areas where the salinity is very low, from 96% to 86% in areas with spraying transport mechanisms of de-icing salt (due to salting roads in winter time) or from 20% to zero in areas with splashing transport mechanisms of de-icing salt.

## Figures and Tables

**Table 1:** Corrosion loss in the corrosion chamber *D_ch_* approximated to the power function and the coefficient of determination R^2^.

Diameter ø	r_ch,first_	b_ch_	R^2^
[mm]	[µm/day]	[–]	[%]
ø6	3.1357	1.0833	99.99
ø10	3.0732	1.0788	99.98
ø14	3.0599	1.0790	99.78
ø25	2.9278	1.0721	99.92
Average	3.05	1.08	99.92

**Table 2:** Basis values for the calculation of the parametric study of the moment resistance in time *M_Rd_(t)* under various conditions in Slovakia.

Values min./max.	Inland Areas without Considering Higher Concentration of *Cl*^−^ (*Cl*^−^ < 3.00 mg/(m^2^·day))		Spraying Transport Mechanisms of De-Icing Salt (*Cl*^−^ = 11.20 mg/(m^2^·day))		Splashing Transport Mechanisms of De-icing Salt (*Cl*^−^ ≥ 760.50 mg/(m^2^·day))	
	b (–)	*r_corr_* (μm/year)	*b* + ∆*b* (-)	*r_corr_* (μm/year)	*b* + ∆*b* (–)	*r_corr_* (μm/year)
Minimum value	0.523	3.00	0.681	10.12	1.00	81.41
Maximum value	0.523	30.00	0.681	38.00	1.00	158.88
